# Cortisol and alpha-amylase as stress response indicators during pre-hospital emergency medicine training with repetitive high-fidelity simulation and scenarios with standardized patients

**DOI:** 10.1186/s13049-015-0110-6

**Published:** 2015-04-08

**Authors:** Bernd Valentin, Oliver Grottke, Max Skorning, Sebastian Bergrath, Harold Fischermann, Daniel Rörtgen, Marie-Therese Mennig, Christina Fitzner, Michael P Müller, Clemens Kirschbaum, Rolf Rossaint, Stefan K Beckers

**Affiliations:** Department of Anesthesiology, RWTH Aachen University Hospital, Aachen, Germany; AIXTRA - Interdisciplinary Centre for Medical Education, RWTH Aachen University Hospital, Aachen, Germany; AIXSIM - Simulation Centre for Anesthesiology, Intensive Care and Emergency Medicine, RWTH Aachen University Hospital, Aachen, Germany; Centre for Learning and Knowledge Management and Information Management in Mechanical Engineering RWTH Aachen University, Aachen, Germany; Department of Medical Statistics, RWTH Aachen University Hospital, Aachen, Germany; Department of Anesthesiology and Intensive Care Medicine, University Hospital Dresden, Dresden, Germany; Institute of Psychology I, University of Technology, Dresden, Germany

**Keywords:** Stress response, Salivary alpha-amylase, Salivary cortisol, High-fidelity simulation, Standardized patients, Post-graduate medical education

## Abstract

**Background:**

In emergency medicine, the benefits of high-fidelity simulation (SIM) are widely accepted and standardized patients (SP) are known to mimic real patients accurately. However, only limited data are available concerning physicians’ stress markers within these training environments.

The aim of this pilot study was to investigate repetitive stress among healthcare professionals in simulated pre-hospital emergency scenarios using either SIM or SPs.

**Methods:**

Teams with one emergency medical services (EMS) physician and two paramedics completed three SIM scenarios and two SP scenarios consecutively. To evaluate stress, salivary cortisol and alpha-amylase were measured in saliva samples taken before, during and after the scenarios.

**Results:**

A total of 14 EMS physicians (29% female; mean age: 36.8 ± 5.0 years; mean duration of EMS-experience: 9.1 ± 5.8 years) and 27 paramedics (11% female; age: 30.9 ± 6.9 years; EMS experience: 8.1 ± 6.0 years) completed the study. Alpha-amylase and cortisol levels did not differ significantly between the two professions. Cortisol values showed a gradual and statistically significant reduction over time but little change was observed in response to each scenario. In contrast, alpha-amylase activity increased significantly in response to every SIM and SP scenario, but there was no clear trend towards an overall increase or decrease over time.

**Conclusion:**

Increases in salivary alpha-amylase activity suggest that both SIM and SP training produce stress among emergency healthcare professionals. Corresponding increases in salivary cortisol levels were not observed. Among physicians in the emergency setting, it appears that alpha-amylase provides a more sensitive measure of stress levels than cortisol.

## Introduction

High-fidelity simulation (SIM) and its benefits are well accepted in undergraduate as well as postgraduate educational settings and SIM is widely used in medicine [1-5]. Human patient simulation represents an alternative: used in teaching environments since 1969, the benefits of this approach have gained acceptance in fields such as aviation training, military war fighter preparation and industry, as well as in medicine [6-10]. Despite the acceptance of these methods, few data exist regarding the occurrence of stress among physicians within these training environments [11,12]. Repetitive stress, which is common in emergency medical care, has been particularly poorly investigated.

Stress is frequently defined as a state of unacceptable divergence between perceived demands and capabilities to adapt [13]. Healthcare professionals in the emergency setting are often confronted with complex situations that lead to stress. Importantly, it has been shown that stress affects the performance of healthcare professionals in different settings [14].

Established methods of measuring stress include heart rate and blood pressure [14]. Biochemical markers of stress are also known to exist in humans. Alpha-amylase is one of the major salivary enzymes in humans, secreted from the salivary gland in response to sympathetic stimuli [15]. Takai et al. reported that alpha-amylase increased by psychological stressors [16]. Cortisol is a stress hormone produced in the adrenal cortex, and its concentration in saliva is strongly correlated with its concentration in blood plasma [17].

This observational study was designed to investigate stress among teams of healthcare professionals in repetitive simulation scenarios using both SIM and standard patients (SP). Salivary levels of cortisol and alpha-amylase were used as markers of stress. This study was part of a broader investigation, where telemedical support in emergency medical services (EMS) was examined [18].

## Materials and methods

### Ethics

This study was approved by the local ethics committee (registration number EK065/09). A standardized leaflet was used to describe the study to each participant and to explain that their performance would be videotaped and evaluated. Participants were then asked to complete a demographic questionnaire and to provide signed consent.

### Setting and interventions

The study was conducted in September 2009 at the interdisciplinary Skillslab facility and the interdisciplinary medical simulation centre of Aachen University Hospital in Germany, and was part of a interdisciplinary research project, where telemedical support in emergency medical services was investigated respective various aspects [18-20]. Five standardized emergency scenarios were designed and tested by an expert committee of five experienced EMS physicians. The scenarios were handled consecutively by teams of 2 paramedics and 1 EMS physician. Lots were used to determine which healthcare professionals would join each team. Realistic environmental conditions were arranged for all scenarios in the simulation centre.

Study participants were equipped with collar microphones (EW 200, Sennheiser, Germany) to record communication with the manikin or the SP, and three cameras in each room (EVI D 70, Sony, Japan) were used to videotape each scenario. Audio and video signals were synchronized.

### Scenario setup: simulation sessions

**Figure 1:**
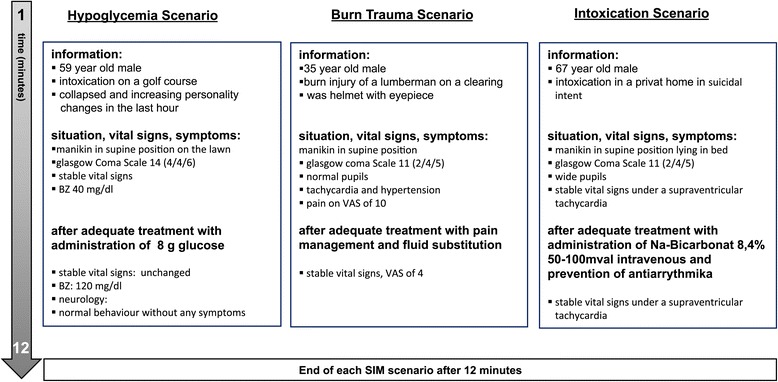
**Details of the SIM scenarios.**

In scenarios 1 to 3 a Human Patient Simulator (HPS™, Medical Education Technologies Inc., Sarasota, FL, USA) was used. Scenario 1 was intended for teams to get familiar with the simulation environment. The manikin used in all three of these scenarios was placed in the supine position on the floor. A simulation instructor, located in the control room, answered all questions addressed to the manikin. Questions about symptoms, past medical history and allergies were answered in a predefined, standardized manner to ensure comparability between the different teams. A second simulation instructor briefed each team and remained present in the simulation room to provide immediate help in case of technical problems. This instructor also described symptoms and appearance that could not be simulated by the manikins, such as skin colour or sweating. A detailed description of the scenarios is provided in Figure 1; each SIM scenario was scheduled to last for 12 minutes, followed by 5 min of a recreation phase, where standardized paper-based documentation was achieved by the physician.

### Scenario setup: standardized patient sessions

**Figure 2:**
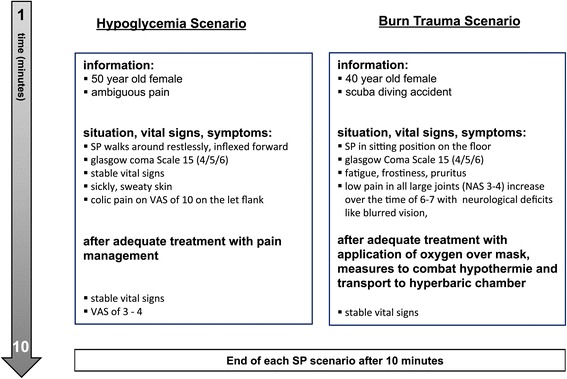
**Details of the SP scenarios.**

Scenarios 4 and 5 were presented by SP with experience from the undergraduate and postgraduate SP programs of the medical faculty. Scripts for the roles were written and approved by the expert committee mentioned above. Questions about symptoms, past medical history and allergies were answered in a predefined, standardized manner by the SP. A detailed description of the scenarios is provided in Figure 2; each SP scenario had a duration of 10 minutes, followed by 5 min of a recreation phase, where standardized paper-based documentation was achieved by the physician. None of the study participants had prior knowledge of the scenarios.

### Methods of measurement: biochemical analyses

**Figure 3:**
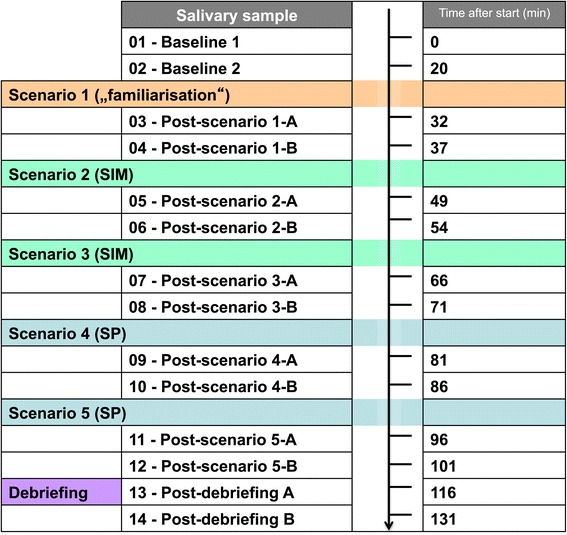
**Overview of study design, showing timepoints at which salivary specimens were taken.**

A total of 14 saliva samples were taken from each participant, to provide levels of alpha-amylase and cortisol before and after each scenario (Figure 3).

Post-scenario A-samples were taken immediately after termination of the scenarios, post-scenario B-samples 5 min later after the recreation phase and consequently right before the start of the following scenario.

Each specimen was centrifuged at 3000 rpm for 5 min (Dresden, Germany). An enzyme kinetic method was then used to determine the concentration of alpha-amylase [21]. The concentration of free cortisol was measured with a commercially available chemiluminescence immunoassay (CLIA; IBL, Germany).

### Data collection and processing

In the absence of similar previous studies, this was an exploratory study and a sample size calculation could not be performed. Instead, the sample size was chosen to be comparable with those of previous studies investigating stress response using biochemical markers [11,12].

### Statistical analysis

Continuous variables following a normal distribution are expressed as mean values ± standard deviation (SD). Non-normally distributed data (amylase and cortisol) are presented as box plots with median, interquartile range (IQR) and range. For both cortisol and alpha-amylase, analysis of covariance (ANCOVA) was conducted for repeated measurements using PROC MIXED software. The following factors were included as covariates: timepoint [1-14], role (EMS physician, paramedic1, paramedic2), group [3-16], gender and age. Time was considered as a repeated factor, and the covariance structure was autoregressive. Due to the exponential distribution of cortisol and alpha-amylase, data were log transformed for the model. A post-hoc analysis was performed to compare the cortisol concentration after the familiarization scenario (timepoint 3) with the levels at subsequent timepoints. Adjusted p-values <0.0038 were considered to be significant. All statistical analysis was carried out with SAS (Version 9.1.3, SAS Institute Inc., Cary, NC, USA).

## Results

### Characteristics of study participants

In total, 16 EMS physicians and 27 paramedics were enrolled for the study. Two EMS physicians were withdrawn due to non-attendance on study day, so 14 groups were evaluated. The dataset of one paramedic was not complete and was eliminated from analysis as well. Among the 14 evaluated EMS physicians, four (29%) were female, the mean age was 36.8 ± 5.0 years, and the mean duration of EMS experience was 9.1 ± 5.8 years. A smaller proportion of the 27 evaluated paramedics were female (11% [n = 3]), they were younger (mean age 30.9 ± 6.9 years) and they had slightly less EMS experience (mean 8.1 ± 6.0 years). Nevertheless, these differences are not considered to be of major importance.

### Salivary alpha-amylase and cortisol levels

**Figure 4:**
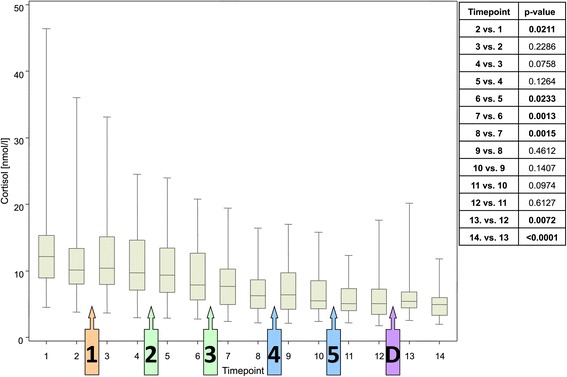
**Cortisol concentration at each assessment timepoint.** Box plots show the median, upper and lower end of the box representing the 1st and 3rd quartile; narrow lines above and below each box represent the range p-values are for paired comparisons between timepoints (level of significance of <0.0038).

Only minor changes in salivary cortisol levels were observed in response to each scenario, although a small decrease in response to the second SIM scenario reached statistical significance (p = 0.0013) (Figure 4). A gradual decrease was apparent over the course of the study, with significant reductions observed in 5 of 13 timepoint comparisons. There was one exception to this trend: a small but statistically significant increase in cortisol after versus before the debriefing. Results of the post-hoc analysis, shown in Table 1, confirm that the decrease over time was statistically significant.

**Table 1:** **Salivary alpha-amylase (**U/ml) **and cortisol levels (**nmol/l) **at each assessment timepoint**

**Time-point**	**Amylase activity**	**Cortisol concentration**	
	**[U/ml]**	**Concentration [nmol/l]**	**P-value for decrease versus time-point 3**
1	38.0 ± 40.7	13.3 ± 7.5	NC
2	51.3 ± 47.2	11.9 ± 6.5	NC
3	54.8 ± 46.3	12.5 ± 6.5	NC
4	35.8 ± 29.0	11.6 ± 5.9	0,0758
5	54.2 ± 45.4	10.8 ± 5.6	0.0134
6	35.2 ± 29.5	9.8 ± 5.0	0.0003
7	52.4 ± 47.4	8.4 ± 4.3	<0.0001
8	41.6 ± 36.2	7.2 ± 3.6	<0.0001
9	60.7 ± 47.2	7.2 ± 3.5	<0.0001
10	33.9 ± 28.4	6.5 ± 3.0	<0.0001
11	51.5 ± 41.4	5.9 ± 2.4	<0.0001
12	44.9 ± 39.5	5.9 ± 3.2	<0.0001
13	61.1 ± 65.5	6.7 ± 3.9	<0.0001
14	58.6 ± 46.5	5.0 ± 2.1	<0.0001

NC, not calculated; NS, not significant.

Data are mean ± standard deviation. Results of post-hoc-analysis comparing cortisol levels at different timepoints are also shown.

**Figure 5:**
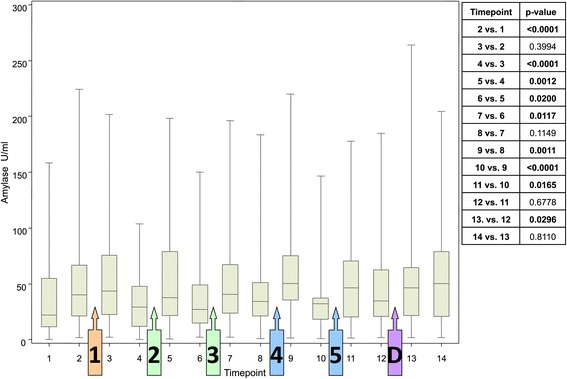
**Alpha-amylase activity at each assessment timepoint.** Box plots show the median, upper and lower end of the box representing the 1st and 3rd quartile; narrow lines above and below each box represent the range. p-values are for paired comparisons between timepoints (level of significance of <0.0038).

A different pattern was observed with alpha-amylase activity. Mean alpha-amylase activity was 38.0 ± 40.7U/ml at baseline (0 min) and increased to 51.3 ± 47.1 U/ml prior to the familiarization scenario (20 min; Table 1). As the study progressed, alpha-amylase activity, showed reproducible and significant increases (p < 0.0038) in response to both SIM and SP scenarios (Figure 5). However, reductions followed each increase, meaning that there was no clear trend towards an increase or decrease over time.

Alpha-amylase activity and cortisol concentration did not differ significantly between professions. Also, there were no significant differences in relation to the gender (p = 0.6275) or age (p = 0.7458) of the participants for cortisol, resp. for alpha-amylase (gender: p = 0.8827; age: p = 0.0433).

## Discussion

The primary aim of this observational study was to investigate stress in multidisciplinary teams of emergency care physicians undertaking repetitive simulation scenarios using SIM and SP. The results show for the first time that alpha-amylase exhibits a characteristic increase in response to simulation scenarios, indicating a degree of stress among the physicians. Increases in alpha-amylase were observed in response to both SIM and SP scenarios. In contrast, cortisol showed no clear changes in response to the simulation scenarios, although a gradual decrease over time was evident.

No significant differences between the two scenario types were observed with respect to concentrations of cortisol or amylase. Interestingly, we also found that neither gender nor age had a significant influence on stress levels. In our study, a pre-hospital setting was chosen, where repetitive management of different cases is routine. Further studies would be needed to ascertain whether our results are applicable to other settings.

High-fidelity simulation is well established in academic settings, but more research is necessary to ascertain its value in medical education [22]. In principle, simulation represents a useful and effective means of promoting and advancing best practice in critical situations. Such training can enhance participant performance and improves physicians’ memory of best practice [23].

Our results are consistent with previous studies showing that simulator training in medicine is able to produce realistic stress [11,12,14]. One study comparing simulated advanced trauma life support training with real-world management of trauma showed a higher degree of stress in response to the simulation [14]. A significant increase in alpha-amylase in response to simulator training has also been shown previously [12]. Müller et al. included two short test scenarios, one performed before and one after a 1-day simulator course. The increase in alpha-amylase in response to the scenario after the 1-day course was smaller than that with the scenario before the course. We did not observe any decrease in the alpha amylase response to later versus earlier scenarios. This could be due to our study design with five scenarios in one day. It is possible that participants were not able to recover adequately between scenarios, or that the learning process induced by the scenario cannot reduce stress in such a short time. Cortisol values in our study were in the same range as those reported by Müller et al. but, unlike in the study by Müller et al., we observed a significant reduction over time in cortisol levels. This suggests that, despite the alpha amylase results, perhaps there was a decrease in stress over time in the current study. Further investigations using additional measurements of stress would be valuable to ascertain the true changes in stress over time among physicians completing five simulation scenarios in one day.

Stress is liable to impair individual as well as team performance in the treatment of patients. For example, it has been shown that medical students with poor stress-coping strategies have reduced laparoscopic dexterity [24]. Similarly, Moorthy et al. investigated the effect of stress-inducing conditions on the performance of laparoscopic task and demonstrated increases in skill- and knowledge-based errors [25]. On the other hand, stress may have some positive effects. Demaria et al. investigated the addition of emotional stressors to training in simulated cardiopulmonary arrest. Participants could remember the events of scenarios in which they felt they had failed, demonstrating that emotional stress can improve memory stages, creation of new memories or the persistence of memories, as well as the ability to recall these memories [23].

In light of these considerations, stress levels induced by training are of significant interest. A degree of stress may be valuable for optimal learning. Future studies could be designed to ascertain the optimal time period between scenarios and which setting – SIM or SP – should be used for the most effective learning. In addition, there is a possibility that training could be used to reduce the stress response to a given situation, potentially translating into improved clinical performance. Our study was not designed to determine the impact of stress on participants’ performance. Future studies investigating whether a reduced stress response after several simulation scenarios has a positive or negative impact on performance would be valuable. A key aim would be to ascertain how best to design training for improvement of performance.

As biochemical markers of stress, salivary alpha-amylase and cortisol seem to have different reaction profiles [26]. Cortisol is a corticosteroid hormone with an influence on memory consolidation in humans [27]; it reacts and recovers less quickly than alpha-amylase [16]. The present findings are consistent with this difference. This is an important difference between our study and others.

There are some limitations to our study. Firstly, the evaluated subjects were not chosen randomly, and this could have introduced a positive selection bias because volunteers often outperform other potential participants [28]. However, because we focused on biochemical measurements instead of performance data, the potential impact of this selection bias on the results should have been reduced. Secondly, for organizational reasons, it was not possible to implement a cross-over-design for rigorous comparison of SIM scenarios with SP scenarios. It was only possible to use one SIM scenario for acclimatization and, as a result, three SIM scenarios were included in the study compared with only two SP scenarios. This could have influenced the results because of possible adaptation during the different types of simulation. Lastly, all data for our study were collected in a single day. In previous studies using levels of cortisol to investigate stress during simulated scenarios, such as that conducted by Müller et al [12], baseline data were typically collected during the day before simulation.

## Conclusion

Over all, this study showed that both SIM and SP training produce stress in teams of paramedics and EMS physicians. Salivary alpha-amylase appears to be a more sensitive marker than salivary cortisol for detecting stress in a simulated pre-hospital environment. Further research is needed to validate these findings in other settings, and to determine how training should be designed for optimal learning and performance improvement.
